# Clinicopathological Significance of Transcription Factor p73 in Breast Cancers: Protein Expression and Transcriptomic Study

**DOI:** 10.3390/biomedicines13102484

**Published:** 2025-10-12

**Authors:** Ahmed Shoqafi, Asmaa Ibrahim, Ayat Lashen, Michael S. Toss, Shatha Alqahtani, Islam Miligy, Mashael Algethami, Amera Sheha, Jennie N. Jeyapalan, Nigel P. Mongan, Andrew R. Green, Emad A. Rakha, Srinivasan Madhusudan

**Affiliations:** 1 Nottingham Biodiscovery Institute, School of Medicine, University of Nottingham, University Park, Nottingham NG7 3RD, UK; ahmed.shoqafi@outlook.sa (A.S.); mrzear1@exmail.nottingham.ac.uk (E.A.R.); 2 Department of Pathology, Nottingham University Hospitals, City Hospital Campus, Nottingham NG5 1PB, UK; 3 Department of Oncology, Nottingham University Hospitals, City Hospital Campus, Nottingham NG5 1PB, UK

**Keywords:** breast cancer, TP53, TP73, pathology, survival, prognosis

## Abstract

**Background**: p73, a member of the p53 family of transcription factors, plays important roles in DNA repair, cell proliferation, angiogenesis, invasion, metastasis, immune evasion, and cytotoxic therapy response. The clinicopathological significance of p73 in breast cancer, particularly in the context of *TP53* mutation, remains largely unknown. **Methods**: Clinicopathological significance of p73 and p53 protein expression was evaluated in 1369 invasive BC and 317 ductal carcinomas in situ (DCIS), including in p53 wild-type or p53 mutant tumours. p73 transcripts and splice variants were investigated in breast cancer genomes (TCGA). **Results**: High cytoplasmic p73 was significantly associated with high tumour grades, high pleomorphism scores, high mitotic scores, high risk Nottingham prognostic index, negative expression of oestrogen receptors (ERs), triple negative phenotypes (all *p* values ≤ 0.01), and poor breast cancer specific survival (BCSS) (*p* = 0.013). In *TP53* mutant breast cancers, high p73 was significantly associated with aggressive histopathological features (all *p* ≤ 0.001) and poor BCSS (*p* = 0.001) but not in p53 wild-type tumours. **Conclusions**: Cytoplasmic p73 may be a marker of aggressive phenotype and worse prognosis, particularly in p53 mutant breast cancer. p73, in conjunction with altered p53 expression, may be involved in breast cancer pathogenesis.

## 1. Introduction

Breast cancer is the most common malignancy among women and a major global health burden, accounting for 25.1% of all female cancers in 2012, with 1.67 million new cases and over 520,000 deaths [1]. Incidence continues to rise, with projections of 3.2 million new cases annually by 2050 [2], and by 2018, approximately 2.1 million cases and 626,679 deaths were reported worldwide [3]. Breast cancer is biologically heterogeneous, comprising subtypes such as Luminal A, Luminal B, HER2-enriched, and triple-negative, each with distinct prognoses and therapeutic responses [4]. Triple-negative breast cancer is often more aggressive and disproportionately prevalent among African American and Hispanic women, highlighting the need for molecular stratification [4]. While incidence is higher in developed countries, mortality remains higher in low-resource regions due to limited healthcare access [4]. Ductal carcinoma in situ (DCIS) and invasive breast cancer (IBC) represent sequential stages of breast cancer progression, with DCIS confined to the ductal system and IBC characterised by stromal invasion and metastatic potential. Although both share common genetic alterations, IBC frequently acquires additional mutations that facilitate invasion and dissemination [5,6]. High-grade DCIS is more likely to progress to IBC, highlighting the contribution of genetic and epigenetic changes in this transition [7]. Clonal analyses further indicate that IBC can evolve directly from DCIS through shared mutational events [8]. There is an ongoing need to understand molecular alterations that drive DCIS to IBC phenotypes.

*TP73* is a member of the *TP53* family of transcription factors [9,10,11]. *TP73* has roles in neurodevelopment, tissue homeostasis, and cancer [10,11,12,13,14,15,16,17]. The structural features of TP73 include three basic functional domains: the transactivation domain (TA), the core DNA-binding domain (DBD), and the oligomerisation domain (OD). TP73 also has a SAM (sterile alpha motif) domain in the C-terminus that promotes the stability of the TP73 protein [11,13,17]. Although TP73 is a structural and functional homologue of *TP53*, it is rarely mutated in solid tumours [10,11,12,13,14,15,16,17]. However, multiple isoforms can be transcribed from the p73 locus [11,13,17]. Alternative splicing at the 5’end generates TA, ΔN, Δ Ex2p73, ΔEx2/3p73, and Δ N’p73 isoforms. At the same time, C-terminal splice variants include α, β, γ, δ, ε, ζ, η, η ∗, η1, and θ isoforms. Δ Ex2p73, ΔEx2/3p73, and ΔN’p73 isoforms partially or entirely lack the transactivation domain and can have a dominant negative (DN) effect over the TA isoform. The DN isoforms include ΔNp73 and ΔN. The TAp73 isoform is a tumour suppressor, but ΔNp73 has oncogenic potential. TP73 knockout mice exhibit complex phenotypes. Total TP73 knockout mice show developmental abnormalities, and TP73+/− heterozygous mice develop cancers. TAp73−/− mice show an increased susceptibility to cancer, but ΔNp73−/− mice do not develop cancers [11]. Taken together, these studies reveal complex biological functions for various TP73 isoforms [11,13,17]. Moreover, TP73 isoforms display an array of protein–protein interactions with nuclear (such as MDM2, YAP1, CDK complex, WT1, Sp1, MCL1, SUMO1, PTEN, MM1, and others) and cytoplasmic proteins (such as NGFR, PKP1, KCK, NEDL2, amphiphysinIIb-1, Wwox, and others) to regulate cellular homeostasis (reviewed in [11,13,17]). Current pre-clinical studies suggest that the TA/DN isoform ratio influences overall cellular phenotype rather than overexpression of a specific TP73 isoform or a specific class of TP73 isoforms [11,13,17].

During cancer pathogenesis, *TP73* may promote genomic instability, pro-proliferative signalling, evasion of growth suppression, activation of invasion and metastasis, angiogenesis, immune evasion, altered cellular energetics, neoneurogenesis, and cytotoxic therapy resistance [10,11,12,13,14,15,16,17]. TP73 dysregulation has been shown in cancer cell lines. TP73 transcripts are overexpressed in breast cancer cell lines. In human cancers, higher levels of TP73 in breast cancer tissue compared to normal breast tissue have been reported [18,19]. However, current research on *TP73* in breast cancer is limited and often contradictory, underscoring the need for further investigation. *TP73* participates in complex regulatory networks that influence tumour progression. Additional regulatory layers include non-coding RNAs and genetic polymorphisms: *TP73*-AS1 has been implicated in promoting proliferation and invasion [20,21], whereas certain polymorphisms, such as G4A, show no clear association with breast cancer risk [22]. Most available studies are based on small cohorts, limiting statistical power and clinical applicability, particularly regarding prognosis and treatment response. To clarify *TP73*’s role, future research should employ larger, more diverse cohorts and integrate analyses of isoform expression, non-coding RNA interactions, and genetic variation.

There is functional cross-talk between p73 and p53 proteins. They share significant structural and functional homology, including conserved DNA-binding and oligomerisation domains [17]. In addition, p73 and p53 proteins regulate overlapping sets of target genes involved in apoptosis, cell cycle control, DNA repair, and genomic stability, thereby functioning as critical tumour suppressors [13,16]. However, unlike *TP53*, which is frequently mutated in breast and other human cancers, *TP73* mutations are rare, and its role is largely thought to be mediated through differential expression of its isoforms [11,18]. Importantly, wild-type p53 can cooperate with p73 to enforce tumour-suppressive pathways, whereas mutant p53 exerts dominant-negative effects by binding and functionally inactivating p73 [12,14,15]. This cross-talk is particularly relevant in breast cancer, where *TP53* mutations are frequent, often in aggressive subtypes such as triple-negative and HER2-enriched tumours [4]. Thus, the biological and clinical effects of *TP73* in breast cancer must be interpreted in the context of *TP53* mutation status, since mutant p53 not only disrupts its own tumour-suppressive functions but also impairs p73 activity, potentially converting p73 from a tumour suppressor into a factor permissive of oncogenesis [12,15].

To address these gaps, we conducted a large-scale analysis of P73/p53 expression in 1,369 invasive breast cancers and 317 DCIS lesions. By integrating mRNA, isoform, and protein-level analyses with *TP53* mutation status and clinicopathologic data, we aimed to clarify the role of *TP73* in breast cancer biology, define its interplay with *TP53*, and assess its potential as a prognostic and therapeutic biomarker. Recent post-2020 transcriptomic studies, including the EMBER platform [23], profiling of triple-negative inflammatory breast cancer, Indian breast cancer transcriptomics [24], multi-country Latin American cohorts [25], and spatial transcriptomics of triple-negative breast cancer [26], further highlight the importance of transcriptomic context in interpreting p73 function and subtype-specific biology.

## 2. Materials and Methods

### 2.1. Patients

We conducted this study in a large series of invasive primary breast cancer (invasive BC) cases consecutively treated at Nottingham University Hospitals (NUHs) between 1986 and 2006. All patients in this cohort were treated in a single institution and have been evaluated in several biomarker studies. Table 1 summarises patient demographics. Patients were treated with standard surgery (mastectomy or wide local excision) and radiotherapy. Patients did not receive systemic adjuvant treatment (AT) before 1989. After 1989, AT was given based on prognostic and predictive factor status, including the Nottingham Prognostic Index (NPI), oestrogen receptor-α (ER-α) status, and menopausal status. Patients with low-risk NPI scores of <3.4 did not receive AT. High-risk pre-menopausal patients with NPI scores of ≥3.4 received classical Cyclophosphamide, Methotrexate, and 5-Fluorouracil (CMF) chemotherapy; patients with ER-α positive tumours were also offered HT. Postmenopausal patients with NPI scores of ≥3.4 and ER-α positivity were treated with HT, and ER-α-negative patients were treated with classical CMF chemotherapy. Median follow-up was 111 months (range: 1 to 233 months). Breast cancer-specific survival (BCSS) and the development of loco-regional and distant metastases (DMs) were maintained on a prospective basis. BCSS was defined as the number of months from diagnosis to the occurrence of BC-related death. DM-free survival was defined as the number of months from diagnosis to the occurrence of DM relapse. Survival was censored if the patient was still alive at the time of analysis, lost to follow-up, or died from other causes. This study was approved by the Yorkshire and the Humber Leeds East Research Ethics Committee (REC Reference: 19/YH/0293, 21 August 2019) under the IRAS Project ID: 266925. Informed consent was obtained from all individuals prior to surgery to use their tissue materials in research. All samples used in this study were pseudo-anonymised and collected prior to 2006 and stored in compliance with the UK Human Tissue Act. The Reporting Recommendations for Tumor Marker Prognostic Studies (REMARK) criteria, recommended by McShane et al. [27], were followed throughout this study.

### 2.2. Tissue Microarray (TMA) and Immunohistochemistry (IHC)

Tumour samples were previously arrayed as Tissue Microarrays (TMAs) using the Grand Master^®^ (3D HISTECH^®^, Budapest, Hungary). For immunohistochemical staining, sections 4 μm thick were cut. Immunohistochemical staining was conducted using the Shandon Sequenza chamber system (REF: 72110017, Biohub, Cheshire, UK) in combination with Novolink Max Polymer Detection System (RE7280-K: 1250 tests, Buffalo Grove, IL, USA) and the Leica Bond Primary Antibody Diluent (AR9352 Buffalo Grove, IL, USA), each used according to the manufacturer’s instructions (Leica Microsystems, Buffalo Grove, IL, USA). Pre-treatment antigen retrieval was performed on the TMA sections using sodium citrate buffer (pH 6.0) and heated for 20 min at 95 °C in a microwave (Whirlpool JT359 Jet Chef 1000W, Peterborough, UK). A set of slides was incubated for 1 h at room temperature with rabbit monoclonal p73 (Abcam, Ab189896, Cambridge, Cambridgeshire, UK)) (dilution 1:500). Immunohistochemical staining for p53 has been described previously [28]. Briefly, monoclonal mouse anti-human p53 [clone DO7] (Novocastra) was used and diluted at a 1:50 ratio in Leica antibody diluent (RE AR9352, Leica, Biosystems, Newcastle Upon Tyne, Tyne and Wear, UK) and incubated for 30 min at room temperature. Immunostaining for p53 showed only nuclear expression. A total of 1369 cases had sufficient invasive tumours (>15% of TMA core) for immunohistochemical staining.

### 2.3. Evaluation of Immunohistochemical Staining

Whole field inspection of the core was scored, and the subcellular localisation of each marker was identified (nuclear, cytoplasmic, or membranous). The intensities of staining within subcellular compartments were assessed and categorised as follows: 0 = no staining, 1 = weak staining, 2 = moderate staining, and 3 = strong staining. The percentage of tumour cells in each category was estimated (0–100%). The Histochemical score (H-score) was calculated by multiplying the staining intensity by the percentage of staining (range: 0–300). A mean H-score of ≤43 was used as the cut-off for p73 cytoplasmic expression. For p53, a 10% cut-off was used as the optimal threshold for dichotomising p53 expression into negative (wild-type) and positive (mutant) tumours. The whole cohort was categorised according to p53 status into wild-type tumours (p53−) and *TP53* mutant tumours (p53+), and clinicopathological variables were investigated for interaction with TP53.

### 2.4. Statistical Analysis

A Chi-squared test was used to evaluate the association with clinical and pathological parameters. All tests were 2-tailed. The Kaplan–Meier method was used for survival rate determination, and the results were compared by the log-rank test. The Statistical Package for the Social Sciences (SPSS, version 22, Chicago, IL, USA) software for Windows was utilised for all analyses. A *p*-value of less than 0.05 was identified as statistically significant.

### 2.5. Transcriptomic Analysis

For *TP73* mRNA expression analysis in normal and breast tumour tissue, we investigated a publicly available RNA-seq gene expression data set (tnmplot.com) [28]. Detailed methods are described by Bartha et al. [28]. Briefly, data processing and analysis features of the TNM-plotter pipeline were developed in R version 3.6.1. Comparison of the normal and tumorous samples was performed by the Mann–Whitney U test. The statistical significance cut-off was set at *p* < 0.01 [28]. Prognostic significance of *TP73* mRNA was evaluated using publicly available gene expression data at http://bcgenex.ico.unicancer.fr/BC-GEM/GEM-Accueil.php?js=1 (accessed on 1 June 2023) [29].

### 2.6. Bioinformatics Analysis

DESeq2 was used to compare RNA expression in TCGA-BRCA samples categorised by the presence or absence of *TP53* coding variants [30]. The VolcaNoseR shinyapp (https://goedhart.shinyapps.io/VolcaNoseR (accessed on 1 June 2023)) was used to prepare a volcano plot to depict differential expression in TCGA-BRCA cases dichotomised based on *TP53* coding variants. The TSV database was also used to compare *P73* transcript level expression in non-malignant vs. malignant breast cancer specimens in the TCGA-BRCA cohort [28,30]. Transcript-level expression estimates for the TCGA-BRCA cohort were accessed from https://osf.io/gqrz9/ (accessed on 1 June 2023), and differential expression in samples with and without TP53 coding variants was identified using DESeq2.

### 2.7. Western Blot Analyses

MCF-10-A, DCIS, MCF-7, T47D, and MDA-MB-231 cell lines were harvested, lysed in RIPA buffer (R0278, Sigma), followed by the addition of protease cocktail inhibitor (P8348, Sigma, UK), phosphatase inhibitor cocktail 2 (P5726, Sigma, UK), and phosphatase inhibitor cocktail 3 (P0044, Sigma, Gillingham, Dorset, UK), and the sample was stored at −20 °C. BCA Protein Assay Kit (23227, Thermo Fisher, Loughborough, UK) was used for protein quantification. Membranes were incubated with primary antibody anti-p73 (1:5000, ab189896, Abcam, Cambridge, Cambridgeshire, UK) at 4 °C overnight, then washed and incubated with β-actin (1:1000, ab8226, Abcam, Cambridge, Cambridgeshire, UK) at room temperature for 1 h. Membranes were later washed and incubated with infrared dye-labelled secondary antibodies (LiCor, Cambridge, Cambridgeshire, UK) [IRDye 800CW donkey anti-rabbit IgG (926-32213) and IRDye 680CW donkey anti-mouse IgG (926-68072)] at a dilution of 1:10,000 for 60 min. The scanning of membranes was performed using the Li-Cor Odyssey Imaging System.

## 3. Results

**Figure 1:**
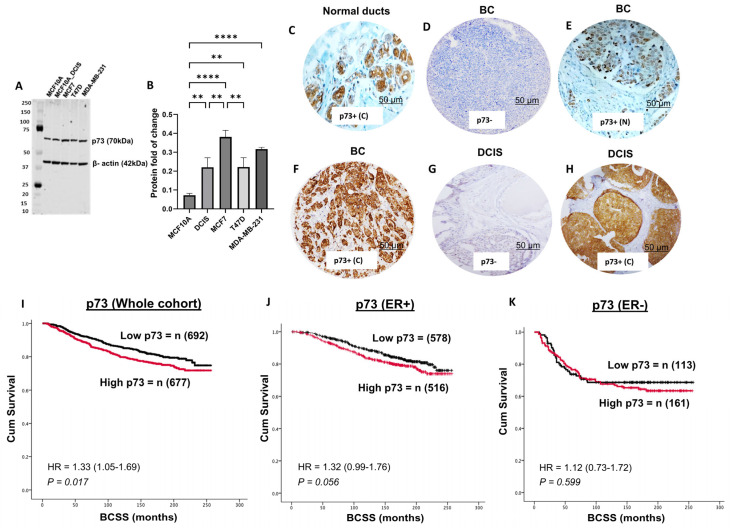
p73 protein expression and breast cancer. (**A**) Western blot of p73 protein expression in a panel of normal (MCF10A), DCIS (MCF10A_DCIS), ER+ (MCF7, T47D), and triple negative (MDA-MB-231) breast cancer cell lines. (**B**) Protein quantification. p73 in breast cancer specimens (imaged at ×20 magnification). (**C**) Normal breast ducts. (**D**) Negative p73 in invasive BC. (**E**) Nuclear p73 in invasive BC. (**F**) Cytoplasmic expression of p73 in invasive BC. (**G**) Negative expression of p73 in DCIS. (**H**) Cytoplasmic p73 in DCIS. (**I**) Cytoplasmic p73 expression and Kaplan–Meier curve for breast cancer specific survival (BCSS) in the whole cohort. (**J**) Cytoplasmic p73 expression and Kaplan–Meier curve for BCSS in ER+ cohort. (**K**) Cytoplasmic p73 expression and Kaplan–Meier curve for BCSS in ER- cohort. ** = *p*-Value < 0.01; **** = *p*-Value < 0.0001; BC = breast cancer; DCIS = ductal carcinoma in situ; C= cytoplasmic; N = nuclear.

We initially evaluated the expression of p73 protein in a panel of normal (MCF10A), DCIS (MCF10A_DCIS), ER+ invasive breast cancer (MCF-7, T47D), and triple-negative (MDA-MB-231) breast cancer cell lines. Normal epithelial cells (MCF10A) have low levels of p73; breast cancer cell lines have high expression of p73 (Figure 1A,B). We proceeded to immunohistochemical evaluation in clinical cohorts.

### 3.1. p73 Protein Expression and Invasive BC

We assessed p73 expression in normal breast ducts of 57 samples and observed cytoplasmic staining only (Figure 1C). In tumours, nuclear expression of p73 was surprisingly rare (Figure 1E), observed in only 14/1369 (1%) of tumours and, therefore, not suitable for clinicopathological association studies. On the other hand, cytoplasmic staining of p73 was seen in 677/1369 (49.4%) tumours (Figure 1F). We proceeded to clinicopathological evaluation in BC. High cytoplasmic p73 levels were significantly associated with features characteristic of aggressive behaviour, including high-grade, pleomorphism, high mitotic index, high-risk Nottingham Prognostic Index (NPI), ER-negative, and triple-negative (TNBC) (all *p*-values ≤ 0.01) (Table 2). In the whole cohort, high p73 was associated with poor outcome in terms of shorter breast cancer-specific survival (BCSS) (*p* = 0.017) (Figure 1I). In ER+ breast cancers, high p73 levels were borderline non-significant for shorter BCSS (*p*= 0.056) (Figure 1J) and non-significant in ER- breast cancers (*p* = 0.599) (Figure 1K).

### 3.2. TP73 mRNA Expression and Invasive BC

**Figure 2:**
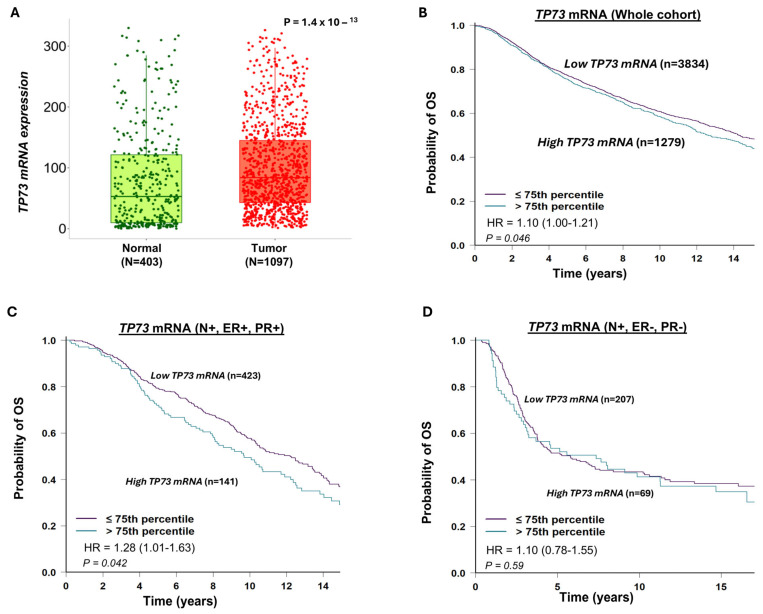
TP73 mRNA expression and breast cancer. (**A**) *TP73* transcripts in normal and breast cancer tissue. The data shows high *TP73* levels in tumour tissue. (**B**) TP73 transcripts and Kaplan–Meier curve for breast cancer specific survival (BCSS) in the whole cohort. The data shows that high *TP73* levels are associated with poor survival in the whole cohort. (**C**) TP73 transcripts and Kaplan–Meier curve for BCSS in node-positive (N+), ER+, and PR- cohorts. The data shows that high *TP73* levels are associated with poor survival in node-positive, ER+ breast cancers. (**D**) TP73 transcripts and Kaplan–Meier curve for BCSS in node-positive (N+), ER-, and PR- cohorts. The data shows no significant associations in node-positive, ER-positive breast cancers. OS = overall survival.

As shown in Figure 2A, *TP73* mRNA expression was significantly higher in breast tumour tissue compared to normal breast tissue (*p* < 0.0001). High *TP73* mRNA expression was significantly linked with poor survival in the whole cohort (Figure 2B), in lymph node+/ER+/PR+ breast cancers (Figure 2C), but not in lymph node+/ER-/PR- breast cancers (Figure 2D).

### 3.3. p53 and Invasive BC

A total of 1601 invasive breast cancers (IBCs) were suitable for p53 immunohistochemical analysis. p53 nuclear positivity was seen in 584/1601 (34.4%) of tumours. p53 positivity was significantly associated with features characteristic of aggressive behaviour, including high-grade, dedifferentiation, pleomorphism, high mitotic index, lymphovascular invasion, high-risk Nottingham Prognostic Index (NPI), HER-2+, ER negative, and triple-negative breast cancers (TNBCs) (all *p*-values ≤ 0.01) (Table 3). In the whole cohort, high p53 expression was associated with a poorer outcome in terms of shorter breast cancer-specific survival (BCSS) (*p* = 0.006) (Figure 2B) but not in ER+(Figure 2C) or ER- tumours (Figure 2D).

### 3.4. p73-p53 Co-Expression in Invasive BC

**Figure 3:**
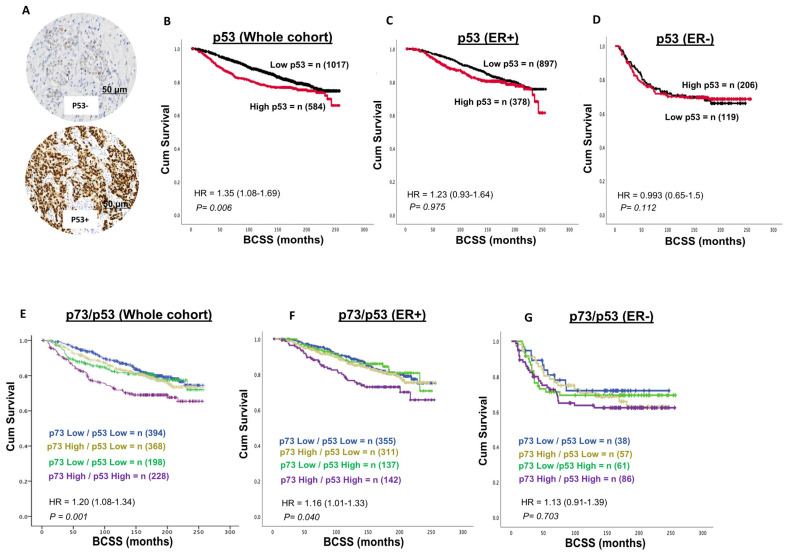
p53-p73 co-expression and breast cancer. (**A**) Representative images of negative and positive p53 staining in breast cancer (×20 magnification). (**B**) p53 expression and Kaplan–Meier curve for breast cancer specific survival (BCSS) in the whole cohort. The data shows that a high p73 level is associated with poor survival in the whole cohort. (**C**) p53 expression and Kaplan–Meier curve for BCSS in the ER+ cohort. The data shows no significant associations. (**D**) p53 expression and Kaplan–Meier curve for BCSS in the ER- cohort. The data shows no significant associations. (**E**) p73-p53 co-expression and Kaplan–Meier curve for breast cancer specific survival (BCSS) in the whole cohort. The data shows that tumours with high p73-p53 expression have poor survival in the whole cohort. (**F**) p73-p53 co-expression and Kaplan–Meier curve for BCSS in ER+ cohort. The data shows that tumours with high p73-p53 expression have poor survival in the ER+ cohort. (**G**) p73-p53 co-expression and Kaplan–Meier curve for BCSS in ER- cohort. The data shows no significant associations. BCSS = breast cancer-specific survival.

A total of 1188 invasive breast cancers (IBCs) were suitable for P73-p53 co-expression analysis. High p73/high p53 expression was observed in 228/1188 (19.1%) tumours and strongly associated with high-grade, dedifferentiation, pleomorphism, high mitotic index, lymphovascular invasion, high-risk Nottingham Prognostic Index (NPI), stage 3 disease, HER-2+, ER negative, PR negative and triple-negative breast cancers (TNBCs) (all *p*-values ≤ 0.01) (Table 3). In the whole cohort, high p73-high p53 co-expression was associated with a poor outcome, characterised by shorter breast cancer-specific survival (BCSS) (*p* = 0.001) (Figure 3E), including in ER+ (*p* = 0.040) (Figure 3F) but not in ER- breast cancer (Figure 3G). Taken together, the data suggests that dysregulation of p73 expression can influence breast cancer pathogenesis and prognosis. To explore whether p73 dysregulation is an early event, we investigated a cohort of 317 non-invasive DCIS.

### 3.5. TP73 Transcripts in BC-TCGA

**Figure 4:**
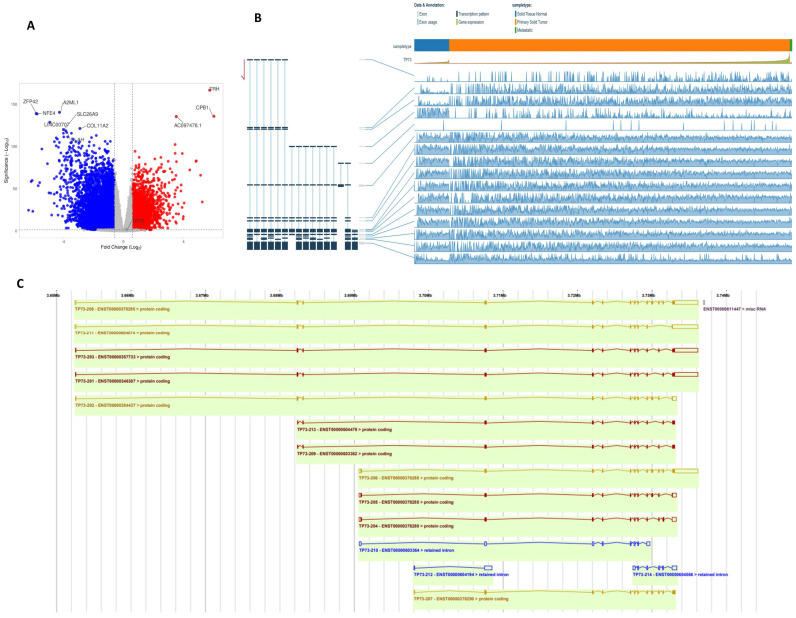
Bioinformatics analysis. (**A**) RNAseq data from the TCGA-BRCA cohort were dichotomised on the basis of *TP53* coding variants. DESeq2 was used to identify 6266 significantly differentially expressed genes (logFC ±≥2, *p* adj < 0.05). (**B**) Expression of *TP73* isoforms in the TCGA-BRCA cohort was assessed in the TSVdb [17]. (**C**) Canonical *TP73* transcripts reported in the ENSEMBL database are presented. Note: Of the 1090 cases included in this cohort, 340 harboured coding variants in the TP53 gene, including stop gain, missense variants, frameshift, splice acceptor, and in-frame variants. No information was available for 109 cases, and the remaining 631 cases had no changes in TP53 coding. Please also see Supplementary Figures S2 and S3 for magnified images.

Using RNAseq data from the TCGA-BRCA cohort, we first dichotomised based on TP53 coding variants. DESeq2 was used to identify 6266 significantly differentially expressed genes (logFC ±≥2, padj < 0.05) (Figure 4A). We then compared *TP73* mRNA expression in the TCGA-BRCA cohort stratified by the presence of *TP53* coding variants. Of the 1090 cases included in this cohort, 340 harboured coding variants in the *TP53* gene, including stop gain, missense variants, frameshift, splice acceptor, and in-frame variants. No information was available for 109 cases, and the remaining 631 cases had no changes in TP53 coding. DESeq2 was used to compare expression in cases with and without *TP53* variants and showed that expression of TP73 was modestly differentially expressed (log2FC = 0.201; padj = 0.021158) in cases harbouring TP53 variants. We then evaluated *TP73* transcript expression in WT TP53 vs. mutant TP53 primary breast cancer. The TSV database was also used to compare TP73 transcript level expression in non-malignant vs. malignant breast cancer specimens in the TCGA-BRCA cohort (https://bmcgenomics.biomedcentral.com/articles/10.1186/s12864-018-4775-x) (accessed on 1 August 2023). Transcript-level expression estimates were determined in the TCGA-BRCA cohort using Kallisto and were accessed from https://osf.io/gqrz9/ (accessed on 1 August 2023). Transcripts with a mean expression <0.1 counts in the cohort were excluded from the analysis. DESeq2 was used to compare global transcript level expression on primary and metastatic breast cancer specimens stratified by the presence or absence of TP53 coding variants. There was no significant difference (±1.5-fold change; padj < 0.05) in any TP73 transcripts expressed in the BRCA cohort (ENST00000378295, ENST00000378288, ENST00000378295, and ENST00000378288). Expression of ENST00000378295 was 1.19-fold higher in primary BCa tumours with normal TP53 expression as compared to those primary tumours harbouring *TP53* coding variants (Figure 4B). Canonical *TP73* transcripts reported in the ENSEMBL database are shown in Figure 4C. We evaluated *TP73* transcript expression in WT *TP53* non-malignant vs. TP53 wild-type primary breast cancer. Expression of the ENST00000378288 transcripts was significantly higher (3.244-fold, *p* < 0.05) in non-malignant breast specimens relative to primary tumours. In contrast, ENST00000378295 P73 transcripts were significantly lower (3.38, padj < 0.05) in non-malignant breast specimens relative to primary tumours. *TP73* transcript expression in non-malignant breast tissue from patients harbouring tumour *TP53* variants vs. TP53 mutant primary breast cancer was then investigated. In patients harbouring TP53 mutations, expression of the ENST00000378295 alone was significantly lower (-2.56-fold change, *p* adj < 0.05) in non-malignant vs. primary breast cancer. Although previous reports identified oncogenic roles for ΔNp73, the current study did not show such an association. We speculate that this may be related to the size of the cohort. Additional bioinformatic studies in larger cohorts with sequencing data will be required to investigate this further. Taken together, the data show a complex expression pattern of *TP73* isoform transcripts in breast cancer.

### 3.6. p73 and p53 Protein Expression in DCIS

Patient demographics are summarised in Table 4. A total of 131/317 (41%) of DCIS showed cytoplasmic expression of p73 (Figure 1E,F), and 59/317 (19%) of DCIS showed nuclear expression of p73. We did not observe any significant association between p73 expression and clinicopathological features (Supplementary Tables S1 and S2) or survival outcomes (Supplementary Figure S1A,B). High nuclear p53 was significantly associated with ER-, PR-, HER2+, and high Ki67 DCIS (Supplementary Table S3). Low nuclear p53 was associated with shorter local recurrence-free interval (Supplementary Figure S1C). When p73 and p53 were combined, we did not observe any significant clinicopathological association (Supplementary Table S4) or survival outcomes (Supplementary Figure S1D,E).

## 4. Discussion

The transcription factor p73 has pleiotropic functions during neurodevelopment, tissue homeostasis, and cancer pathogenesis [9,10,11,12,13,14,15,16,30]. Given the multiple splice variants of p73 with distinct biological functions [11,13,17], the precise role of p73 in breast cancer pathogenesis remains undefined. Here, we have conducted the largest study to date of p73 expression in clinical breast cancers. At the transcriptomic level, *TP73* mRNA expression was high in tumour tissue compared to normal tissue and linked with shorter survival outcomes. In the TCGA-BC cohorts harbouring *TP53* variants, *TP73* transcripts were differentially expressed. Low-abundance transcripts (<100 counts) were filtered, and differentially expressed transcripts in breast cancer patients stratified on the basis of the presence or absence of p53 coding variants were identified by DESeq2. While this analysis identified 17493 differentially expressed transcripts, two expressed *TP73* transcripts (ENST00000378295.8, ENST00000378288.8) were not altered by p53 variants under the criteria defined (>1.5-fold change, pAdj < 0.05). We identified 6266 differentially expressed genes (>2-fold change, padj < 0.05) in TCGA primary breast cancer cases, dichotomised based on the presence or absence of *TP53* variants. This represents ~10% of the genes 60486 analysed. We then investigated *TP73* splice variants in the TCGA-BC cohort. We did not observe any difference in the expression of *TP73* splice variants expressed in the BRCA cohort (ENST00000378295, ENST00000378288, ENST00000378295, and ENST00000378288). Expression of ENST00000378295 was modestly higher in primary BC tumours with normal *TP53* as compared to those primary tumours harbouring *TP53* coding variants. Interestingly, the ENST00000378288 transcripts were significantly higher in non-malignant breast specimens relative to primary tumours. In contrast, ENST00000378295 *TP73* transcripts were significantly lower in non-malignant breast specimens relative to primary tumours. Taken together, our data demonstrate a complex expression pattern of *TP73* isoform transcripts in breast cancer.

Although complex, the overall biological effect of p73 isoforms is likely influenced by the TA/DN isoform ratio as opposed to the overexpression of a specific p73 isoform or a specific class of p73 isoforms in cells [11,13,17]. Therefore, we proceeded to immunohistochemical evaluation of p73 in a large cohort of breast cancer patients. In invasive breast cancer, we surprisingly observed only cytoplasmic p73 staining. Nuclear p73 staining was rare. High cytoplasmic p73 protein level was associated with aggressive phenotypes and poor survival. Wwox, a tumour suppressor protein, has previously been shown to be involved in cytoplasmic sequestration of p73 [31]. Whether the functional association between Wwox and p73 could account for the cytoplasmic staining of P73 observed in the current study is unknown. Detailed mechanistic studies would be required to confirm this hypothesis. Wwox has been shown to inhibit the apoptotic activity of p73 [31]. In addition, the cytoplasmic localisation of p73 and its interaction with other proteins, including HCK and amphiphysin IIb-1, may also inhibit apoptotic activity of p73 [32] and could contribute to aggressive phenotypes observed in the current study. The cohort spanning 1986–2006 was selected for protein analysis due to its comprehensive clinical annotation, long-term follow-up, and availability of well-preserved archival tissue suitable for biomarker analysis. Importantly, the cohort followed standardised treatment protocols, as detailed in Section 2, which helps to minimise variability and supports the robustness of the findings. While treatment practices have evolved, our study focused primarily on molecular associations rather than treatment outcomes, and this is acknowledged as a limitation. A further limitation of our study is that we did not investigate individual p73 splice variants due to the non-availability of antibody clones that recognise specific p73 splice variants. We have used a Rabbit monoclonal anti-p73 antibody (Abcam clone -ab189896) for the IHC studies. The antibody, as indicated by the manufacturer (Product datasheet: Anti-p73 antibody [EPR18409(T)(MIX)] ab189896), has been shown to recognise the C-terminal fragment of p73, which contains amino acids 380-636. The data imply that the antibody recognises all splice variants, and the levels may indicate the total p73 expression in cells. However, a limitation is that we cannot rule out the possibility of cross-reaction with other structurally similar proteins, such as p63 or even unrelated antigens with shared epitopes.

In DCIS, p73 did not influence clinical outcome, suggesting a role in the pathogenesis of invasive cancers only. In tumours with mutant p53, we show that high p73 was not only associated with aggressive pathology but also shorter survival in patients. However, a limitation of our study is the assumption that *TP53* mutations would result in overexpression of p53 protein, causing higher p53 staining intensity, and wild-type protein would stain with low intensity. However, this categorisation strategy leaves out the category of mutations that lead to null expression, and these tumours may have been grouped with tumours with wild-type *TP53*. In a previous study, the expression levels of TAp73 and DeltaTAp73 (DeltaEx2p73, DeltaEx2/3p73, and DeltaNp73) were evaluated in 60 breast cancer samples. Both suppressor and oncogenic isoforms of p73 were significantly co-upregulated in tumours in that study [33]. In another small study, similarly, p73 overexpression was observed in a panel of breast cancer cell lines and human breast cancer tissue [34]. However, neither of these studies investigated the clinicopathological significance of p73 and its impact on survival in the context of p53 variants. p73 overexpression has also been described in other solid tumours, including ovarian cancer, liver cancer, bladder cancer, prostate cancer, and colorectal cancers [11,13,30]. In contrast, loss of p73 has been shown in pancreatic cancers [35]. It has been shown previously that the ratio between TA and δN splice variant expression can influence biology and prognosis. Accordingly, δNp73 overexpression correlated with aggressive features and poor prognosis in neuroblastoma, prostate, head and neck, and cervical cancers [11,13,30]. In the current study on breast cancer, however, we did not observe any significant differences in splice variant expression in the large TCGA-BC data set.

Our study demonstrates that cytoplasmic p73 expression is associated with aggressive tumour features and poor outcomes in invasive breast cancer, particularly in tumours harbouring *TP53* mutations. However, the underlying mechanism of cytoplasmic sequestration of p73 remains unknown and is a limitation of our study. This observation suggests that subcellular localisation modulates p73 tumour-suppressive function, consistent with prior reports of cytoplasmic sequestration mediated by Wwox, HCK, and amphiphysin IIb-1, which inhibit p73 transcriptional and apoptotic activity [31,32]. In *TP53*-mutant tumours, these interactions may exacerbate loss of tumour suppression, highlighting a critical cross-talk between p53 and p73 in determining tumour behaviour [12,14,15]. Interestingly, p73 expression was not prognostic in DCIS, suggesting that dysregulation may be a later event in tumour progression. This raises the possibility that p73, particularly its cytoplasmic sequestration, could serve as a marker for transition from in situ to invasive disease, warranting longitudinal validation. Understanding the mechanisms underlying cytoplasmic retention, including interactions with Wwox and other regulatory proteins, may provide insight into the stepwise progression of breast cancer [31,32]. From a translational perspective, cytoplasmic p73 could serve as a prognostic biomarker, especially in *TP53*-mutant tumours. Immunohistochemical assessment of p73, with attention to subcellular localisation, could complement standard clinical markers such as ER, PR, HER2, Ki67, and p53, enabling more precise risk stratification. Moreover, functional inactivation of p73 in the cytoplasm identifies a potential therapeutic target: agents that restore nuclear localisation or transcriptional activity of p73 could reinstate tumour-suppressive programmes, particularly in tumours lacking functional p53 [9,10,11,13,18]. Isoform-specific effects could further guide targeted interventions, such as inhibiting oncogenic ΔTAp73 isoforms or enhancing tumour-suppressive TAp73 activity [17]. Integration of large-scale transcriptomic data further contextualises these findings. The EMBER platform [23] and other post-2020 studies [24,25,26] highlight heterogeneity in breast cancer subtypes and transcriptomic signatures, reinforcing the relevance of TP73 isoform analysis and its interplay with *TP53* status. Such integration allows for more precise predictions of tumour behaviour and identification of patients who may benefit from p73-targeted therapies. Overall, this study contributes to the field by linking p73 biology directly to *TP53* status, emphasising the importance of isoform balance, localisation, and functional interactions in breast cancer pathogenesis. By combining mRNA, isoform, and protein-level analyses, our work advances mechanistic understanding and establishes a framework for precision oncology strategies that exploit *TP53*–TP73 pathways. Future research should focus on mechanistic validation of cytoplasmic p73 regulation, functional assessment of isoform-specific activity, and development of therapeutic approaches to restore p73 tumour-suppressive function, ultimately improving outcomes for patients with *TP53*-mutant breast cancers. In this study, *TP53* mutation status was inferred using immunohistochemistry (IHC), with strong nuclear staining indicating mutation and weak or absent staining suggesting a wild-type status. While this approach is widely used, it has inherent limitations. Tumours harbouring null mutations, truncating variants, or mutations leading to unstable p53 protein may be misclassified as wild-type, potentially confounding the assessment of p73–p53 interactions [36]. As we did not have TP53 sequencing data to define TP53 mutations in our cohort, this may influence the interpretation of p73-p53 interactions.

## 5. Conclusions

Here, we show that high cytoplasmic p73 is linked with aggressive pathology, such as high tumour grade, high pleomorphism scores, high mitotic scores, high-risk Nottingham Prognostic Index, ER negativity, triple-negative phenotype, and poor survival, particularly in *TP53* mutant breast cancers. Our data suggest that cytoplasmic p73 could be a useful stratification tool. Integrating immunohistochemical assessment of p73 into clinical workflows could complement existing markers, enhancing precision oncology strategies. Future studies should focus on validating cytoplasmic p73 as a prognostic biomarker, elucidating the mechanistic basis of its cytoplasmic sequestration, and developing therapies that restore its tumour-suppressive function. Large-scale transcriptomic resources [28,29,30] provide a valuable framework for such investigations, offering subtype-specific insights and enabling more personalised therapeutic approaches.

## Figures and Tables

**Table 1:** Demographics of invasive breast cancer (IBC) cohort.

Parameters	N %
Tumour size	
<2 cm	852 (58%)
≥2 cm	628 (42%)
Grade	
1	253 (17%)
2	549 (37%)
3	678 (46%)
Tubular formation	
1	119 (8%)
2	456 (31%)
3	905 (61%)
Pleomorphism	
1	44 (3%)
2	500 (34%)
3	936 (63%)
Mitosis	
1	647 (44%)
2	294 (20%)
3	539 (36%)
Histological tumour type	
No special type (NST)	923 (62%)
Lobular	132 (9%)
Other special types	73 (5%)
NST mixed	352 (24%)
Lymphovascular invasion	
Absent	1040 (70%)
Present	440 (30%)
Lymph node status	
Negative	937 (63%)
Positive	543 (37%)
Nottingham Prognostic Index	
Good prognostic group	491 (33%)
Moderate prognostic group	769 (52%)
Poor prognostic group	220 (15%)
ER status	
ER-	347 (24%)
ER+	1128 (76%)
PgR status	
Negative	601 (41%)
Positive	857 (59%)
HER2 status	
Negative	1264 (86%)
Positive	199 (14%)
Ki67 index	
Low	533 (48%)
High	580 (52%)
Molecular classes	
Luminal A	478 (38%)
Luminal B	442 (36%)
HER2+	87 (7%)
Triple negative	235 (19%)
Menopausal Status	
Pre-menopausal	540 (36%)
Postmenopausal	940 (64%)
Age: 50 years	
<50	481 (32%)
≥50	999 (68%)

**Table 2:** Cytoplasmic p73 protein expression and clinicopathological associations.

Parameters	p73 Cytoplasmic Expression		R^2^ *p* Value
	Low (N%)	High (N%)	
Tumour size			
<2 cm	440 (52%)	400 (48%)	2.922
≥2 cm	252 (48%)	277 (52%)	0.087
Grade			
1	114 (53%)	100 (47%)	15.241
2	302 (56%)	237 (44%)	<0.0001
3	276 (45%)	340 (55%)	
Tubular formation			
1	52 (53%)	46 (47%)	0.508
2	207 (51%)	196 (49%)	0.776
3	433 (50%)	435 (50%)	
Pleomorphism			
1	8 (44%)	10 (56%)	13.117
2	231 (58%)	166 (42%)	0.001
3	453 (47%)	501 (53%)	
Mitosis			
1	362 (55%)	298 (45%)	11.729
2	135 (50%)	134 (50%)	0.003
3	195 (44%)	245 (56%)	
Histological tumour type			
No special type (NST)	424 (48%)	460 (52%)	16.248
Invasive lobular carcinoma (ILC)	69 (62%)	42 (38%)	0.012
Mixed NST and ILC	45 (54%)	38 (46%)	
Mixed NST and special type	14 (37%)	24 (63%)	
Pure special tumour type	14 (70%)	6 (30%)	
Metaplastic carcinoma	2 (40%)	3 (60%)	
Tubular and tubular mixed	124 (54%)	104 (46%)	
Lymphovascular invasion			
Absent	506 (52%)	474 (48%)	1.624
Present	186 (48%)	203 (52%)	0.203
Lymph node status			
Negative	438 (52%)	408 (48%)	1.33
Positive	254 (49%)	269 (51%)	0.249
Nottingham Prognostic Index			
Good	261 (56%)	205 (44%)	
Moderate	322 (47%)	357 (53%)	8.531
Poor	109 (49%)	115 (51%)	0.014
Nodal stage			
1	438 (52%)	408 (48%)	
2	193 (51%)	184 (49%)	5.06
3	61 (42%)	85 (58%)	0.08
ER status			
ER-	113 (41%)	161 (59%)	11.78
ER+	578 (53%)	516 (47%)	0.001
PgR status			
Negative	269 (48%)	291 (52%)	2.34
Positive	418 (52%)	382 (48%)	0.126
HER2 status			
Negative	592 (50%)	590 (50%)	0.634
Positive	99 (53%)	87 (47%)	0.426
Triple negative (TN)			
Non-TN	614 (53%)	546 (47%)	17.738
TN	71 (37%)	123 (63%)	<0.0001
Molecular classes			
Luminal	578 (53%)	516 (47%)	
HER2 enriched	36 (55%)	30 (45%)	17.811
TNBC	71 (37%)	123 (63%)	<0.0001
Menopausal status			
Pre	218 (47%)	247 (53%)	3.786
Post	474 (52%)	430 (48%)	0.052
Age			
<50	193 (45%)	233 (55%)	6.8
≥50	499 (53%)	444 (47%)	0.009

**Table 3:** p53 protein expression in breast cancer.

Parameters	TP53 Expression		R^2^ *p* Value
	Low (N%)	High (N%)	
Tumour size			
<2 cm	634 (65%)	343 (35%)	2.03
≥2 cm	383 (61%)	241 (39%)	0.154
Grade			
1	187 (78%)	52 (22%)	
2	474 (75%)	157 (25%)	128.278
3	356 (49%)	375 (51%)	<0.0001
Tubular formation			
1	84 (76%)	26 (24%)	
2	324 (68%)	153 (32%)	17.066
3	609 (60%)	405 (40%)	<0.0001
Pleomorphism			
1	17 (74%)	6 (26%)	
2	373 (81%)	85 (19%)	92.017
3	627 (56%)	493 (44%)	<0.0001
Mitosis			
1	581 (77%)	173 (23%)	
2	187 (60%)	125 (40%)	127.902
3	249 (46%)	286 (54%)	<0.0001
Histological tumour type			
No special type (NST)	598 (57%)	459 (43%)	
Invasive lobular carcinoma (ILC)	108 (86%)	17 (14%)	
Mixed NST and ILC	68 (73%)	25 (27%)	
Mixed NST and special type	27 (66%)	14 (34%)	73.222
Pure special tumour type	13 (72%)	5 (28%)	<0.0001
Metaplastic carcinoma	3 (60%)	2 (40%)	
Tubular and tubular mixed	200 (76%)	62 (24%)	
Lymphovascular invasion			
Absent	744 (65%)	395 (35%)	5.505
Present	273 (59%)	189 (41%)	0.019
Lymph node status			
Negative	637 (65%)	348 (35%)	1.357
Positive	380 (62%)	235 (38%)	0.244
Nottingham Prognostic Index			
Good	392 (73%)	141 (27%)	
Moderate	494 (61%)	321 (39%)	40.584
Poor	131 (52%)	121 (48%)	<0.0001
Nodal stage			
1	637 (65%)	348 (35%)	
2	291 (64%)	164 (36%)	4.905
3	89 (56%)	71 (44%)	0.086
ER status			
ER-	119 (37%)	206 (63%)	127.185
ER+	897 (70%)	378 (30%)	<0.0001
PgR status			
Negative	354 (54%)	300 (46%)	43.509
Positive	660 (70%)	279 (30%)	<0.0001
HER2 status			
Negative	914 (66%)	469 (34%)	29.329
Positive	101 (47%)	114 (53%)	<0.0001
Ki67 index			
Low	458 (76%)	149 (24%)	60.599
High	319 (54%)	272 (46%)	<0.0001
Molecular classes			
Luminal	897 (70%)	378 (30%)	
HER2 enriched	25 (32%)	54 (68%)	127.187
TNBC	89 (38%)	146 (62%)	<0.0001
Menopausal status			
Pre	337 (60%)	225 (40%)	4.732
Post	680 (65%)	359 (35%)	0.03
Age			
<50	296 (58%)	215 (42%)	10.148
≥50	721 (66%)	369 (34%)	0.001

**Table 4:** Demographics of ductal carcinoma in situ (DCIS) cohort.

Categories	N (%)
Age at time of diagnosis	
<50	87 (27%)
≥50	230 (73%)
Tumour size	
≤2 cm	133 (42%)
>2 cm	181 (58%)
Tumour grade	
Low grade	47 (15%)
Intermediate grade	85 (27%)
High grade	185 (58%)
Molecular subtype	
Luminal A	128 (54%)
Luminal B	44 (19%)
HER2 enriched	37 (16%)
Triple negative	27 (11%)
Comedo type necrosis	
Absent	113 (36%)
Present	204 (64%)
ER Status	
Negative	68 (25%)
Positive	207 (75%)
PR status	
Negative	115 (42%)
Positive	160 (58%)
HER2 status	
Negative	226 (78%)
Positive	64 (22%)
Recurrence	
No recurrence	279 (88%)
Recurrence	38 (12%)
Ki67 expression	
Low (≤14)	192 (76%)
High (>14)	60 (24%)

## Data Availability

Raw data will be made available upon reasonable request.

## Supplementary Materials

The following supporting information can be downloaded at https://www.mdpi.com/article/10.3390/biomedicines13102484/s1, Supplementary Table S1: Cytoplasmic TP73 and DCIS. Supplementary Table S2: Nuclear TP73 and DCIS. Supplementary Table S3: Nuclear TP53 and DCIS. Supplementary Table S4: TP73/TP53 co-expression and DCIS. Supplementary Figure S1: Cytoplasmic TP73 expression and survival. Supplementary Figure S2: Bioinformatics analysis. Enlarged image of expression of TP73 isoforms in the TCGA-BRCA cohort was assessed in the TSVdb. Please see main Figure 4B for further details. Supplementary Figure S3: Bioinformatics analysis. Enlarged image of Canonical TP73 transcripts reported in the ENSEMBL database are presented.

## Abbreviations

The following abbreviations are used in this manuscript:

BCSS	Breast cancer-specific survival
TA	Transactivation domain
DCIS	Ductal carcinoma in situ
NPI	Nottingham Prognostic Index
IHC	Immunohistochemistry
