# Supplementary material for: Clinicopathological Significance of Transcription Factor p73 in Breast Cancers: Protein Expression and Transcriptomic Study
Source: Biomedicines. 2025 Oct 12;13(10):2484. doi: 10.3390/biomedicines13102484 (PMC12561529; doi:10.3390/biomedicines13102484)
Supplement: Supplementary file 1 [file biomedicines-13-02484-s001.zip › biomedicines-3826040-supplementary.pdf]

Supplementary Table S1. Cytoplasmic TP73 and DCIS.

Parameters	*TP73 Cytoplasmic Expression*		*X2*
	Low (N%)	*High (N%)*	*p value*
**Age 50 Years**			
≤ 50	43 (23%)	44 (34%)	4.231
> 50	143 (77%)	87 (66%)	**0.04**
**Tumour size**			
≤ 2cm	80 (44%)	53 (41%)	0.229
> 2cm	104 (56%)	77 (59%)	0.632
**Grade**			
Low	28 (15%)	19 (15%)	
Intermediate	48 (26%)	37 (28%)	0.233
High	110 (59%)	75 (57%)	0.89
**Comedo Type Necrosis**			
No	66 (36%)	47 (36%)	0.005
Yes	120 (64%)	84 (64%)	0.943
**Residual_Tumor**			
No	37 (47%)	17 (36%)	1.369
Yes	42 (53%)	30 (64%)	0.242
**ER Status**			
ER-	43 (26%)	25 (22%)	0.588
ER+	120 (74%)	87 (78%)	0.443
**PgR Status**			
Negative	67 (41%)	48 (43%)	
Positive	95 (59%)	65 (57%)	
**HER2 Status**			
Negative	135 (79%)	91 (76%)	0.25
Positive	36 (21%)	28 (23%)	0.617
**Ki67**			
Low	115 (78%)	77 (74%)	0.452
High	33 (22%)	27 (26%)	0.501
**Molecular Classes With Ki-67**			
Luminal A	76 (55%)	52 (54%)	
Luminal B	23 (17%)	21 (22%)	3.737
HER2 Enriched	20 (14%)	17 (17%)	0.291
TNBC	20 (14%)	7 (7%)	
**Recurrence**			
No	163 (88%)	116 (88%)	0.061
Yes	23 (12%)	15 (12%)	0.805

Supplementary Table S2. Nuclear TP73 and DCIS.

Parameters	*TP73 Nuclear Expression*		*X2*
	Low (N%)	*High (N%)*	*p value*
**Age 50 Years**			
≤ 50	66 (26%)	21 (36%	2.417
> 50	192 (74%)	38 (64%)	0.12
**Tumour size**			
≤ 2cm	106 (41%)	27 (47%)	0.513
> 2cm	150 (59%)	31 (53%)	0.474
**3 Tier Grade**			
Low	39 (15%)	8 (13%)	
Intermediate	68 (26%)	17 (29%)	0.193
High	151 (59%)	34 (58%)	0.908
**Comedo Type Necrosis**			
No	93 (36%)	20 (34%)	0.097
Yes	165 (64%)	39 (66%)	0.756
**Residual_Tumor**			
No	46 (44%)	8 (36%)	0.459
Yes	58 (56%)	14 (64%)	0.498
**ER Status**			
ER-	51 (23%)	17 (32%)	1.905
ER+	171 (77%)	36 (68%)	0.168
**PgR Status**			
Negative	92 (41%)	23 (46%)	0.439
Positive	133 (59%)	27 (54%)	0.507
**HER2 Status**			
Negative	194 (82%)	32 (62%)	9.9
Positive	44 (18%)	20 (38%)	**0.002**
**Ki67**			
Low	157 (75%)	35 (80%)	0.331
High	51 (25%)	9 (20%)	0.565
**Molecular Classes With Ki-67**			
Luminal A	108 (56%)	20 (46%)	
Luminal B	36 (19%)	8 (18%)	5.674
HER2 Enriched	25 (13%)	12 (27%)	0.129
TNBC	23 (12%)	4 (9%)	
**Recurrence**			
No	226 (88%)	53 (90%)	0.227
Yes	32 (12%)	6 (10%)	0.634

Supplementary Table S3. Nuclear TP53 and DCIS.

Parameters	*TP53 Expression*		*X2*
	Low (N%)	***High (N%)***	***p value***
**Age 50 Years**			
≤ 50	73 (27%)	56 (25%)	0.263
> 50	197 (73%)	168 (75%)	0.608
**Tumour size**			
≤ 2cm	131 (49%)	99 (45%)	0.754
> 2cm	139 (51%)	123 (55%)	0.385
**3 Tier Grade**			
Low	39 (15%)	24 (11%)	
Intermediate	79 (29%)	50 (22%)	5.872
High	152 (56%)	150 (67%)	0.053
**Comedo Type Necrosis**			
No	101 (37%)	74 (33%)	1.023
Yes	169 (63%)	150 (67%)	0.312
**Residual_Tumor**			
No	49 (45%)	38 (40%)	0.352
Yes	61 (55%)	56 (60%)	0.553
**ER Status**			
ER-	53 (21%)	65 (31%)	6.692
ER+	204 (79%)	144 (69%)	**0.01**
**PgR Status**			
Negative	87 (34%)	103 (49%)	10.407
Positive	167 (66%)	107 (51%)	**0.001**
**HER2 Status**			
Negative	218 (83%)	151 (71%)	10.277
Positive	44 (17%)	62 (29%)	**0.001**
**Ki67**			
Low	187 (82%)	135 (70%)	8.465
High	41 (18%)	58 (30%)	**0.004**
**Molecular Classes With Ki-67**			
Luminal A	133 (61%)	78 (43%)	
Luminal B	34 (16%)	43 (23%)	13.974
HER2 Enriched	26 (12%)	34 (19%)	**0.003**
TNBC	24 (11%)	28 (15%)	
**Recurrence**			
No	237 (88%)	202 (90%)	0.713
Yes	33 (12%)	22 (10%)	0.398

Supplementary Table S4. TP73/TP53 co-expression and DCIS.

Parameters	*Cytoplasm_TP73 and TP53 Co-expression*				***X2***
	TP73- / TP53-	***TP73+ / TP53+***	***TP73+ / TP53-***	***TP73- / TP53+***	***p value***
**Age 50 Years**					
≤ 50	23 (29%)	17 (33%)	17 (32%)	14 (26%)	0.613
> 50	57 (71%)	35 (67%)	37 (68%)	39 (74%)	0.893
**Tumour size**					
≤ 2cm	37 (46%)	18 (35%)	23 (43%)	21 (40%)	1.654
> 2cm	43 (54%)	33 (65%)	31 (57%)	32 (60%)	0.647
**3 Tier Grade**					
Low	14 (17%)	6 (12%)	9 (17%)	6 (11%)	
Intermediate	27 (34%)	13 (25%)	16 (29%)	11 (21%)	6.023
High	39 (49%)	33 (63%)	29 (54%)	36 (68%)	0.421
**Comedo Type Necrosis**					
No	31 (39%)	20 (38%)	18 (33%)	18 (34%)	0.642
Yes	49 (61%)	32 (62%)	36 (67%)	35 (66%)	0.887
**Residual Tumor**					
No	16 (50%)	6 (33%)	10 (42%)	9 (36%)	1.759
Yes	16 (50%)	12 (67%)	14 (58%)	16 (64%)	0.624
**ER Status**					
Negative	18 (23%)	17 (35%)	7 (14%)	15 (30%)	7.222
Positive	59 (77%)	31 (65%)	45 (86%)	35 (70%)	0.065
**PgR Status**					
Negative	27 (35%)	25 (51%)	15 (30%)	25 (49%)	7.017
Positive	50 (65%)	24 (49%)	35 (70%)	26 (51%)	0.071
**HER2 Status**					
Negative	64 (81%)	31 (63%)	45 (85%)	38 (73%)	8.065
Positive	15 (19%)	18 (37%)	8 (15%)	14 (27%)	0.045
**Ki67**					
Low	61 (84%)	33 (72%)	35 (78%	31 (65%)	6.13
High	12 (16%)	13 (28%)	10 (22%)	17 (35%)	0.105
**Molecular Classes With Ki-67**					
Luminal A	46 (65%)	19 (42%)	27 (63%)	19 (42%)	
Luminal B	9 (12%)	9 (20%)	10 (23%)	12 (27%)	14.93
HER2 Enriched	9 (12%)	12 (27%)	5 (12%)	8 (18%)	0.093
TNBC	8 (11%)	5 (11%)	1 (2%)	6 (13%)	
**Recurrence**					
No	69 (86%)	47 (90%)	46 (85%)	46 (87%)	0.727
Yes	11 (14%)	5 (10%)	8 (15%)	7 (13%)	0.867

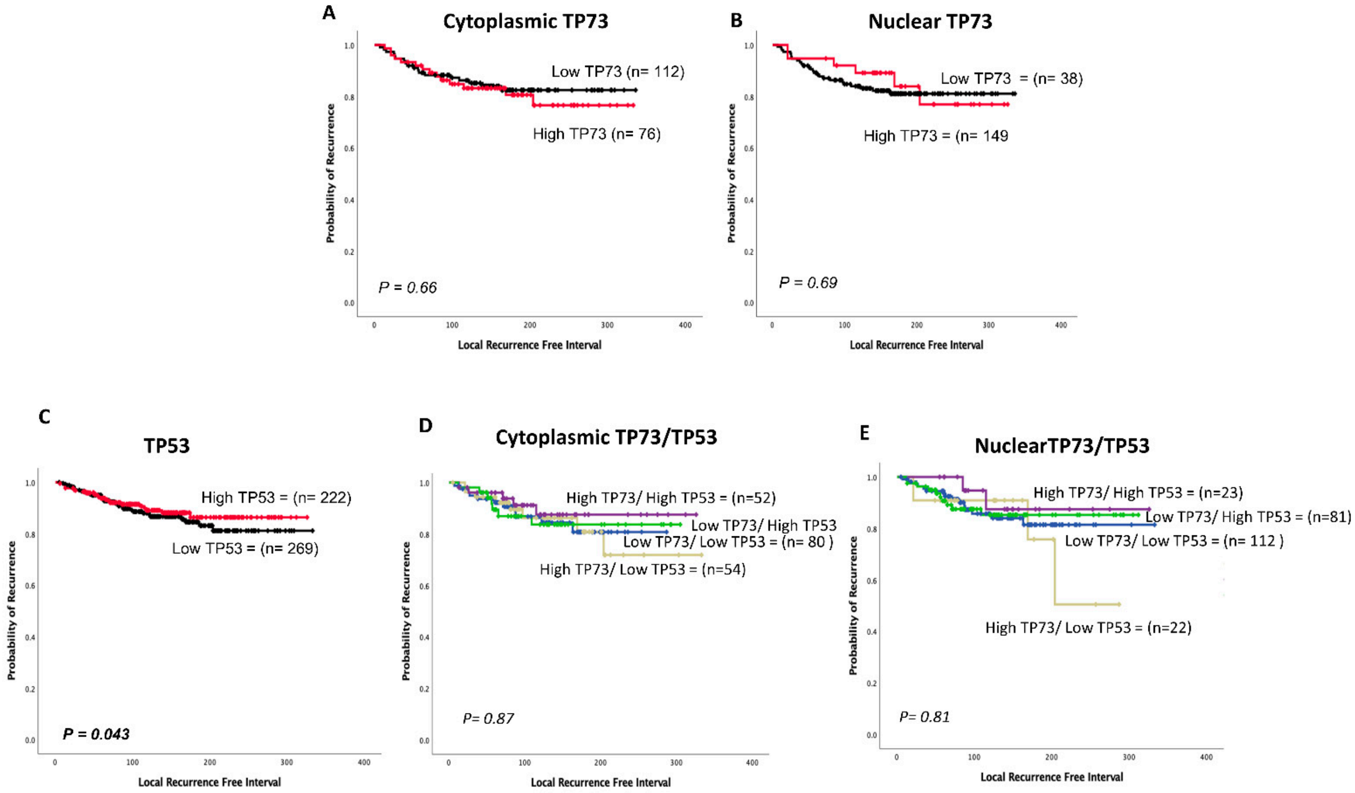

Supplementary Figure S1: (A) Cytoplasmic TP73 expression and Kaplan-Meier curve for local recurrence in DCIS cohort. (B) Nuclear TP73 expression and Kaplan-Meier curve for local recurrence in DCIS cohort. (C) Nuclear TP53 expression and Kaplan-Meier curve for local recurrence in DCIS cohort. (D) Cytoplasmic TP73/Nuclear TP53 expression and Kaplan-Meier curve for local recurrence in DCIS cohort. (E) Nuclear TP73/Nuclear TP53 expression and Kaplan-Meier curve for local recurrence in DCIS cohort.

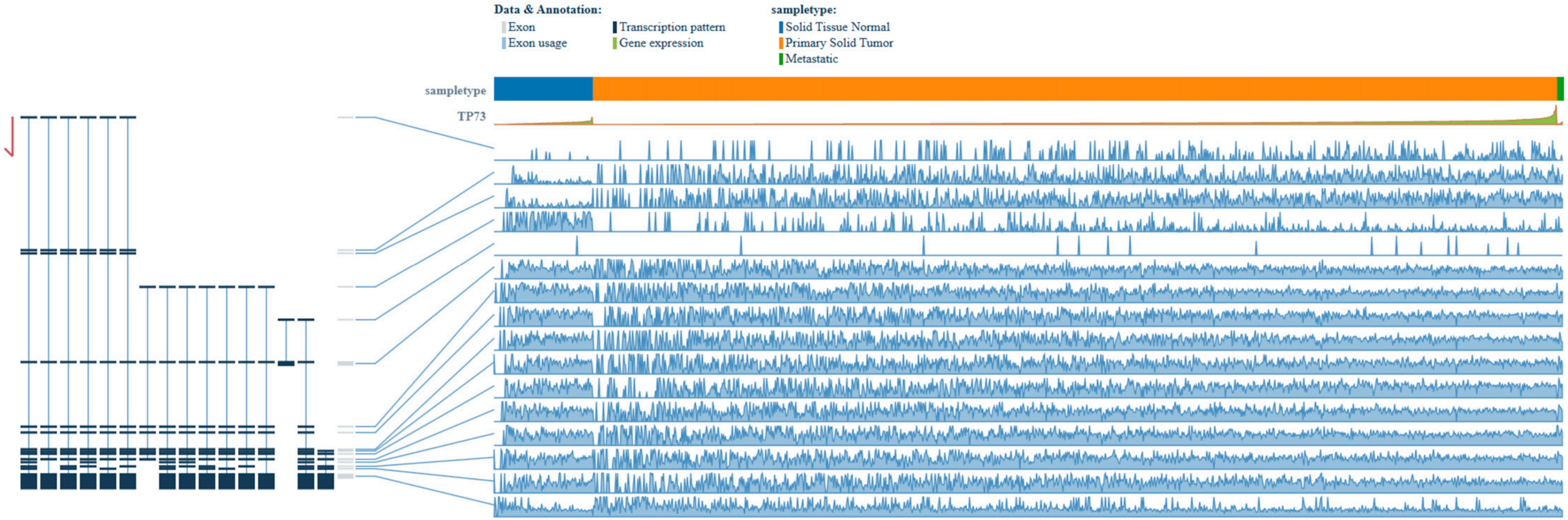

Supplementary Figure S2: Bioinformatics analysis. Enlarged image of expression of TP73 isoforms in the TCGA-BRCA cohort was assessed in the TSVdb. Please see main Figure 4B for further details.

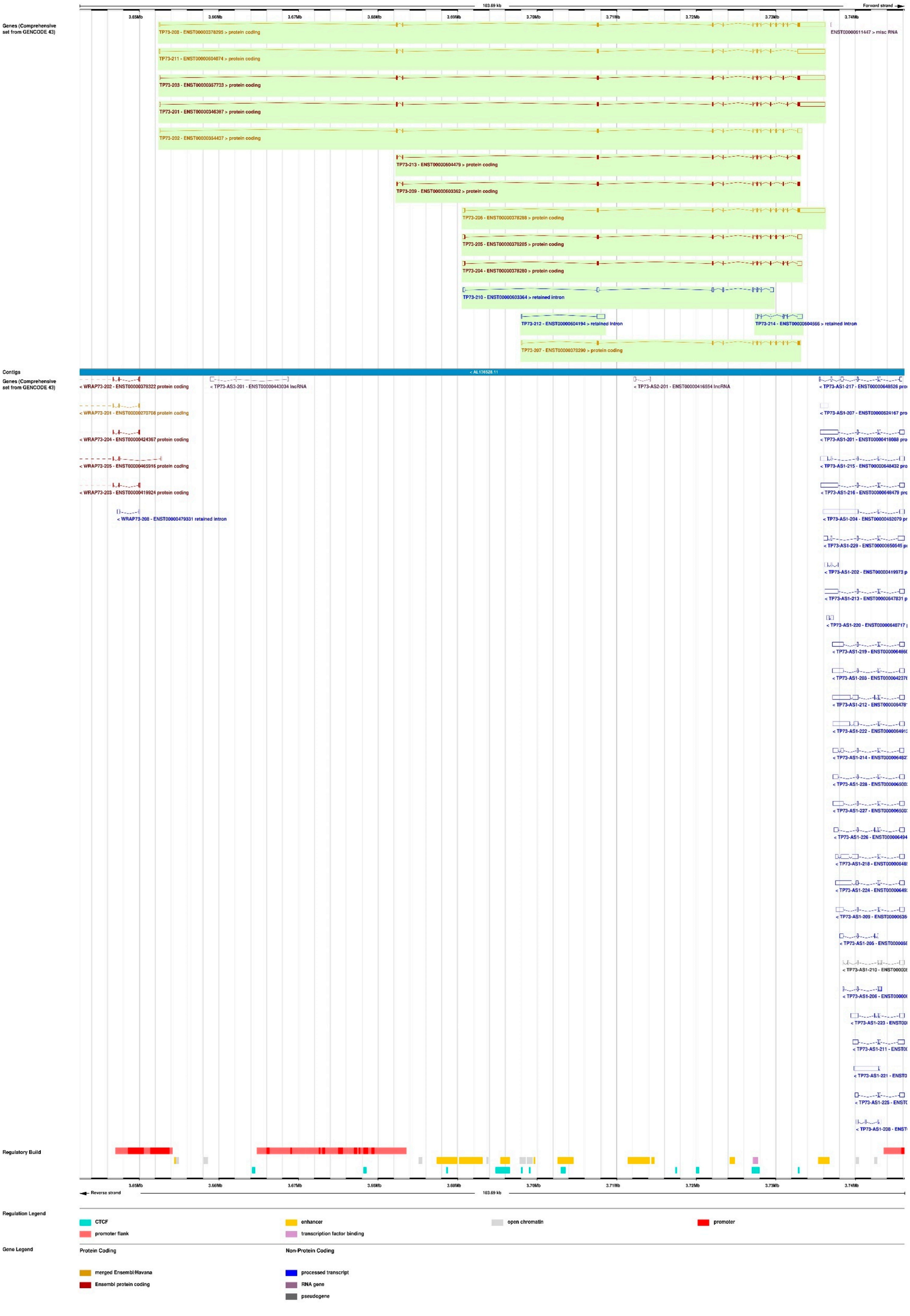
103.69 kb
Forward strand
3.65Mb
3.66Mb
3.67Mb
3.68Mb
3.69Mb
3.70Mb
3.71Mb
3.72Mb
3.73Mb
3.74Mb
Genes (Comprehensive set from GENCODE 43)
TP73-208 - ENST00000378295 > protein coding
ENST00000611447 > misc RNA
TP73-211 - ENST00000604074 > protein coding
TP73-203 - ENST00000357733 > protein coding
TP73-201 - ENST00000346387 > protein coding
TP73-202 - ENST00000354437 > protein coding
TP73-213 - ENST00000604479 > protein coding
TP73-209 - ENST00000603362 > protein coding
TP73-206 - ENST00000378288 > protein coding
TP73-205 - ENST00000378285 > protein coding
TP73-204 - ENST00000378280 > protein coding
TP73-210 - ENST00000603364 > retained intron
TP73-212 - ENST00000604194 > retained intron
TP73-214 - ENST00000604566 > retained intron
TP73-207 - ENST00000378290 > protein coding
Contigs
< AL136528.11
Genes (Comprehensive set from GENCODE 43)
< WRAP73-202 - ENST00000378322 protein coding
< TP73-AS3-201 - ENST00000443034 lncRNA
< TP73-AS2-201 - ENST00000416554 lncRNA
< WRAP73-201 - ENST00000270708 protein coding
< WRAP73-204 - ENST00000424367 protein coding
< WRAP73-205 - ENST00000465916 protein coding
< WRAP73-203 - ENST00000419924 protein coding
< WRAP73-208 - ENST00000479331 retained intron
Regulatory Build
Reverse strand
Regulation Legend
CTCF
enhancer
open chromatin
promoter
promoter flank
transcription factor binding
Gene Legend
Protein Coding
Non-Protein Coding
merged Ensembl/Havana
Ensembl protein coding
processed transcript
RNA gene
pseudogene

Supplementary Figure S3: Bioinformatics analysis. Enlarged image of Canonical TP73 transcripts reported in the ENSEMBL database are presented.
